# A longitudinal, retrospective registry-based validation study of RETTS©, the Swedish adult ED context version

**DOI:** 10.1186/s13049-022-01014-4

**Published:** 2022-04-15

**Authors:** Sara C. Wireklint, Carina Elmqvist, Bengt Fridlund, Katarina E. Göransson

**Affiliations:** 1 Emergency Department, Region Kronoberg, Växjö, Sweden; 2 Department of Research and Development, Region Kronoberg, Sigfridsvägen 5, 352 57 Växjö, Sweden; 3 Department of Health and Caring Sciences, and Centre of Interprofessional Collaboration Emergency Care (CICE), Linnaeus University, Växjö, Sweden; 4 Centre of Interprofessional Collaboration Within Emergency Care (CICE), Linnaeus University, Växjö, Sweden; 5 Caring Sciences, School of Health and Welfare, Dalarna University, Falun, Sweden

**Keywords:** Emergency Department—Emergency Service, Hospital, Rapid emergency triage and treatment system—RETTS©, Sweden, Triage—emergency medical services, Validity—reproducibility of results

## Abstract

**Background:**

Triage and triage related work has been performed in Swedish Emergency Departments (EDs) since the mid-1990s. The Rapid Emergency Triage and Treatment System (RETTS©), with annual updates, is the most applied triage system. However, the national implementation has been performed despite low scientific foundation for triage as a method, mainly related to the absence of adjustment to age and gender. Furthermore, there is a lack of studies of RETTS© in Swedish ED context, especially of RETTS© validity. Hence, the aim the study was to determine the validity of RETTS©.

**Methods:**

A longitudinal retrospective register study based on cohort data from a healthcare region comprising two EDs in southern Sweden. Two editions of RETTS© was selected; year 2013 and 2016, enabling comparison of crude data, and adjusted for age-combined Charlson comorbidity index (ACCI) and gender. All patients ≥ 18 years visiting either of the two EDs seeing a physician, was included. Primary outcome was ten-day mortality, secondary outcome was admission to Intensive Care Unit (ICU). The data was analysed with descriptive, and inferential statistics.

**Results:**

Totally 74,845 patients were included. There was an increase in patients allocated red or orange triage levels (unstable) between the years, but a decrease of admission, both to general ward and ICU. Of all patients, 1031 (1.4%) died within ten-days. Both cohorts demonstrated a statistically significant difference between the triage levels, i.e. a higher risk for ten-day mortality and ICU admission for patients in all triage levels compared to those in green triage level. Furthermore, significant statistically differences were demonstrated for ICU admission, crude as well as adjusted, and for adjusted data ten-day mortality, indicating that ACCI explained ten-day mortality, but not ICU admission. However, no statistically significant difference was found for the two annual editions of RETTS© considering ten-day mortality, crude data.

**Conclusion:**

The annual upgrade of RETTS© had no statistically significant impact on the validity of the triage system, considering the risk for ten-day mortality. However, the inclusion of ACCI, or at least age, can improve the validity of the triage system.

**Graphical Abstract:**

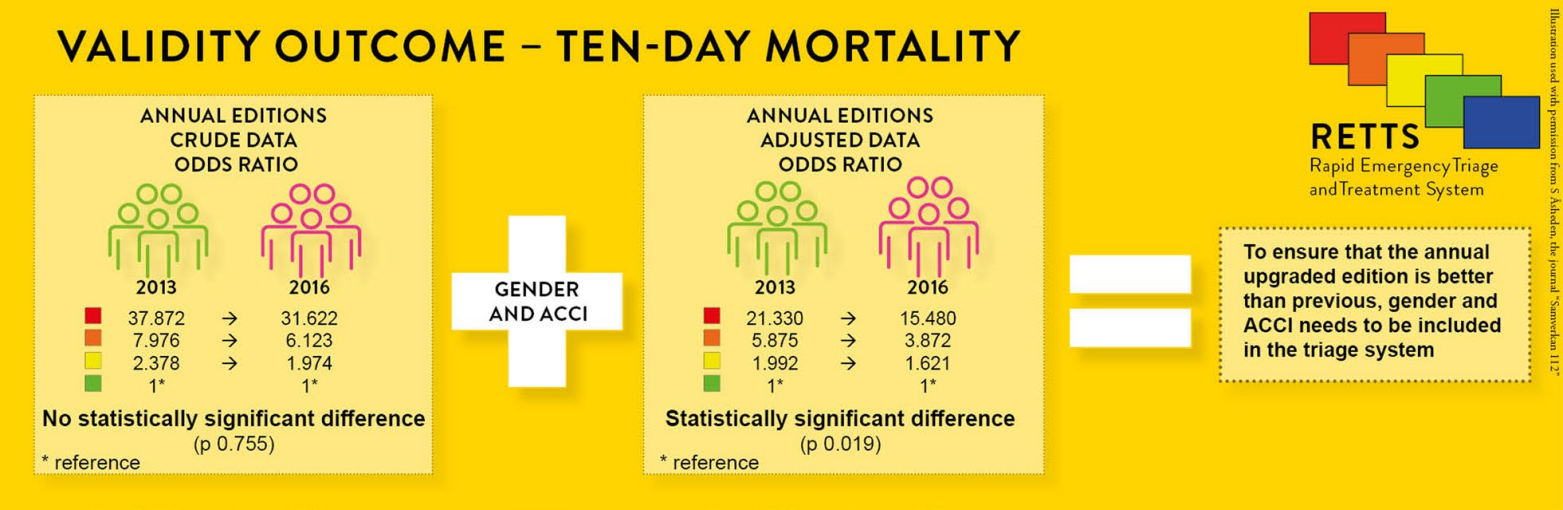

## Background

During the last decades many Emergency Departments (EDs) report crowding problems [1]. One intervention to address ED crowding is triage, defined as sorting patients according to their medical needs using a triage scale [2]. Most commonly known scales are the Australasian Triage Scale (ATS) [3], the Canadian Triage and Acuity Scale (CTAS) [4, 5], the Manchester Triage System (MTS) [6] and the Emergency Severity Index (ESI) [7], which all have demonstrated validity [8–10].

**Fig. 1:**
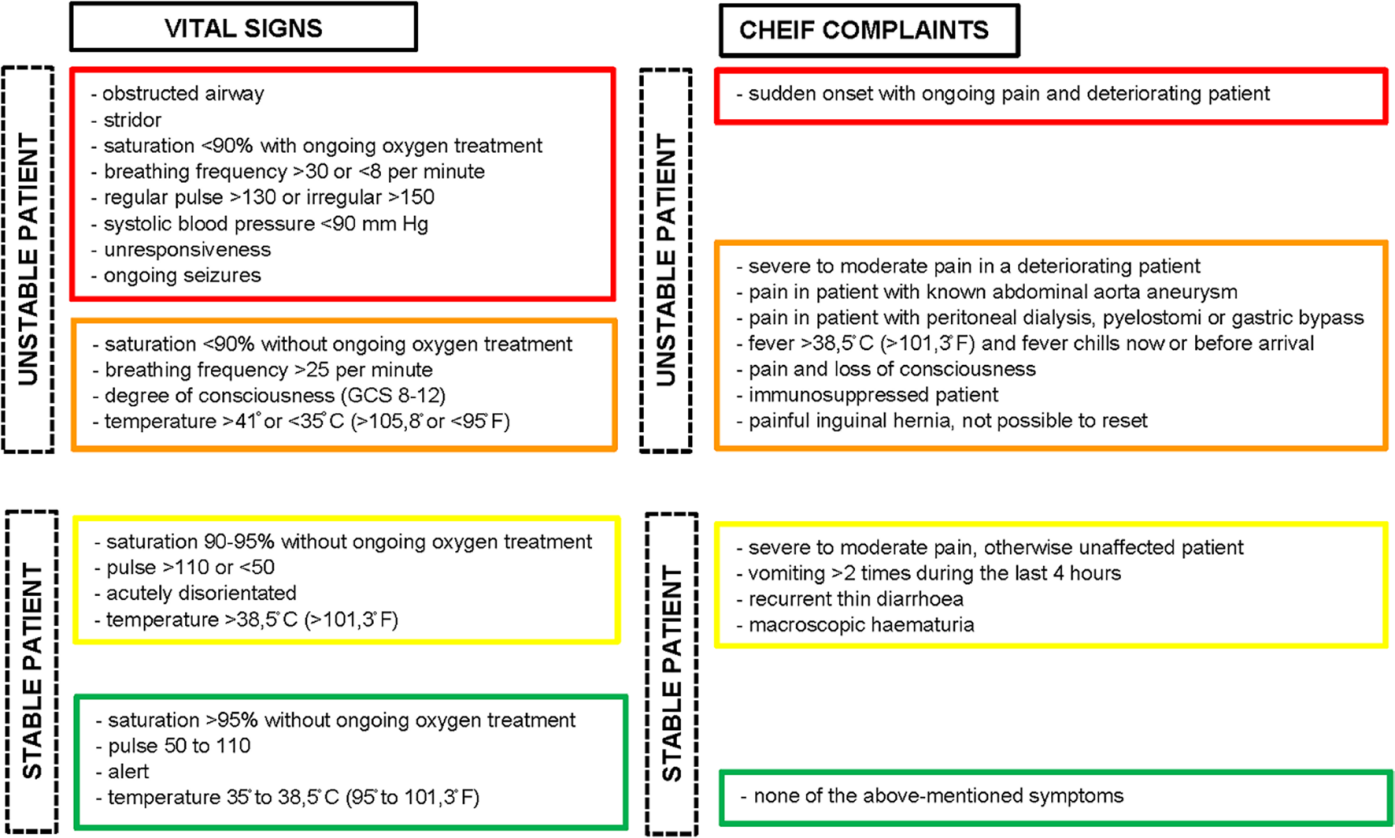
Cut off levels due to vital signs and ESS no. 6, *abdominal pain,* according to the manual for RETTS©, edition 2014

In Sweden, triage was spread across the country in the mid-2000s, initially with MTS, and thereafter with nationally developed triage scales such as Adaptive Process Triage (ADAPT) and the Medical Emergency Triage and Treatment System (METTS) [11]. These Swedish triage scales spread to adjacent countries; a modified Danish version of ADAPT, Danish Emergency Process Triage (DEPT) [12], and METTS in Norway [13]. In Sweden, METTS subsequently became the Rapid Emergency Triage and Treatment System (RETTS©) [14], as well as in Norway [15]. A version called Rapid Emergency Triage and Treatment System—Hospital Unit West (RETTS-HEV) was implemented in Denmark [16]. RETTS© is a process-orientated five-level triage system, using the triage levels of red, orange, yellow, green and blue, in declining priority of acuity, i.e. red is the highest priority, etc. The system combines chief complaints, called *Emergency Symptoms and Signs* (ESS), with vital signs (VS), and both ESS and VS have cut-off levels that indicate levels of acuity (Fig. 1). The most pronounced deviation in either VS or ESS determines the triage level. RETTS© uses a defined boundary for unstable and stable patients, i.e. a boundary that determines whether or not a patient can wait for physician examination without their condition deteriorating, although without stipulating any specific time frames except for the red triage level. Furthermore, there are versions of RETTS© for Adult, Paediatric, Psychiatric and Pre-hospital triage [14] and it is continuously updated in order to improve the assessment [17] but it is not mandatory to apply each upgrade.

In Sweden and Norway RETTS© is the most commonly used triage system [15, 18]. Validity studies of RETTS© in Norway (paediatric version) and Denmark (adult version) report the system to valid based on significant associations between hospitalisation, and admission to intensive care for children with high triage levels [19] and a strong association between triage levels and admission to hospital and in-hospital mortality [20].

In Sweden, RETTS© has mainly been studied in a pre-hospital context. A pre-hospital paediatric version demonstrated a moderate sensitivity, since two-thirds of the patients triaged with red or orange triage level later were identified as non-emergent [21]. Furthermore, a version of RETTS© adult, demonstrated a higher sensitivity (73%) to detecting time-sensitive conditions, but lower specificity (54%) compared to NEWS and NEWS2 [22]. In the Swedish ED context, a study of METTS (i.e. the predecessor of RETTS), demonstrated validity through significantly longer hospital LOS, and in-hospital mortality, associated with higher triage levels [23]. According to a review of triage and patient flow in Swedish EDs by the Swedish Council of Health Technology Assessment (SBU) in 2010, the scientific evidence for triage was insufficient mainly related to the absence of adjustment for age and gender, and that the scientific basis for METTS was deficient [24]. In summary, despite the nationwide establishment of triage in general, and RETTS© in particular, we have only been able to identify one study about the validity of RETTS© regarding adult patients in a Swedish ED context. Hence, there is a knowledge gap regarding RETTS© in a Swedish ED context, particularly regarding its validity. The aim of the study was to determine the validity of RETTS©.

## Methods

### Design and setting

This study had a longitudinal retrospective register-based design using cohort data from a healthcare region in Southern Sweden comprising two EDs. Together, these EDs had approximately 42,000 visits annually. At both settings, physicians had on-call duty whilst RNs and assistant nurses were employed at the EDs working shifts, rotating between medical specialities and emergency rooms. RETTS© edition 1.0 was implemented at the EDs in 2011, as well as a four-hour target, which is why the fifth RETTS© (blue) triage level was redundant. At the implementation of RETTS© edition 1.0, a 4-h education session was performed, while a two-hour update session followed the upgrading to the 2014 edition. Staff employed after 2014 only received the 2-h update session. The major difference between the two editions used in the study was the increased number of symptoms for describing red and orange triage levels in 40% of the ESS in the 2014 edition.

### Data sources and collection

**Fig. 2:**
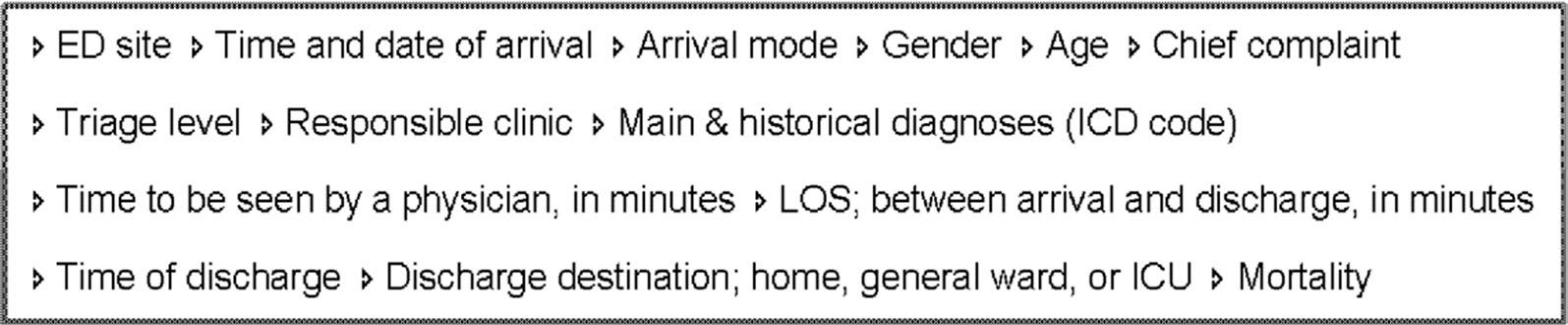
Variables extracted from the computerised medical record system

The two editions of RETTS© (1.0 and 2014) had been applied 2 years respectively prior to data collection. The data (Fig. 2) were collected from the administrative system connected to the general computerised medical records through the *Department of healthcare data analytics and quality assessments* in the region in 2018. Inclusion criteria were adult patients (≥ 18 years) who visited either of the two EDs during 2013 or 2016, and who were seen by a physician.

Since the computerised medical record system is connected to the national population register, all requested data were retrieved from this system, including mortality status.

The initial data comprised N = 75,930 contacts for the two sites together: 2013; n = 36,777 and 2016; n = 39,153. However, 1085 contacts were found to be invalid data due to duplicates, misregistration and consultations, i.e. refers to hospitalized patients in need of assessment from a physician stationed at the ED, leaving n = 74,845 distributed as n = 36,323 from 2013, and n = 38,522 from 2016.

Since the extracted data were not from an established quality register, an algorithm for data control was constructed by the research group: 15 variables multiplied by ten patients, multiplied by two cohorts. This yielded 300 randomly selected charts, extracted from the data set by the *Department of healthcare data analytics and quality assessments*, which became subject to chart audits. The research group decided that if less than 20% of the variables contained incorrect data, the data should be regarded as acceptable, and that an initial quality test would be performed halfway through the charts. After reviewing 160 charts (80 per year) of the 300 predetermined charts, the quality test demonstrated that seven (47%) of the 15 variables included were 100% correct. The remaining eight (53%) variables were correct on average 98% (ranging between 93 and 99%). Based on the results, the research group determined that the data were of high quality and refrained from further auditing of the remaining predetermined charts.

### Analyses

Demographic data were analysed using descriptive statistics. Since patients ≤ 18 years were excluded, the data became skewed and reported with median and interquartile range (IQR). Pearson’s chi-square test was used for comparison between the annual cohorts. The triage data were analysed using multiple logistic regression, both crude and adjusted for the predictors of co-morbidity and age according to the age-combined Charlson comorbidity index (ACCI) [25], together with gender. Female gender, green triage level, zero ACCI points and the year 2013 were used as references in the calculations. Ten-day mortality was the primary outcome and admission to an intensive care unit (ICU) the secondary outcome. A crude version was calculated based on triage levels, both years together, and divided by years, followed by an equal calculation based on the adjusted data. ACCI was applied as described by Sundararajan et al. [26], i.e. with ICD 10 implemented (“Appendix”). The results were reported by odds ratio (OR) and a 95% confidence interval (C.I.), with two-sided *p* < 0.05, considered as statistically significant. Receiver operating characteristic (ROC) curves were calculated and reported in combination with area under curve (AUC), with values from 0.7 to 0.8 considered as acceptable, 0.8 to 0.9 as excellent and > 0.9 as outstanding [27]. The ROC and AUC calculations were performed on crude data, all triage levels and both years together. Missing data occurred only in the variables arrival mode and time to physician, and, since they are not included in the validity analyses, were treated as missing. All statistics were performed using IBM’s SPSS, version 27. Since the study was a total population study, a power calculation was redundant.

## Results

### Background characteristics

A total of 74,845 patients met the inclusion criteria and are therefore included in the study (Table 1). The yearly census had increased by 2199 (3%) patients between 2013 and 2016.There was no statistically significant difference between the two cohorts regarding age. In both annual cohorts the most commonly allocated triage level was yellow. The number of patients with comorbidity or high age, i.e. having one or more ACCI points, decreased per triage level. Patients who left without being seen by a physician increased from 2013 to 2016 by more than 100%. However, they did not exceed 0.5% of the total. In 2016, patients visit at the EDs were in median 27 min longer, compared to 2013. The most common discharge destinations in both annual cohorts were the patients’ home. There was a decrease in admission rates, both to general wards and ICUs, although there was an increase in patients assessed as unstable (red and orange triage level) during the same period. Of all 74,845 ED patients, almost one fifth (18%) were assessed as being unstable. ED mortality was low in both annual cohorts.

**Table 1:** Description of the two emergency departments and characteristics of patients visiting in 2013 and 2016

	2013	2016	Total
ED visits	36,323	38,522	74,845
Age years, median (IQR)	61 (36)	61 (37)	61 (37)
Gender n (%)			
Female	18,489 (51)	19,193 (50)	37,682 (50)
Male	17,834 (49)	19,329 (50)	37,163 (50)
Arrival mode			
Ambulance^a^	10,737 (30)	10,943 (28)	21,680 (29)
Walking^a^	21,396 (59)	24,295 (63)	45,691 (61)
Missing	4190 (11)	3284 (9)	7474 (10)
Triage level n (%)			
Red	1011 (3)	1357 (4)	2386 (3)
Orange	4965 (14)	6307 (16)	11,272 (15)
Yellow	21,374 (59)	23,469 (61)	44,843 (60)
Green	8973 (25)	7371 (19)	16,344 (22)
No. of patients with one or more ACCI points at triage, per triage level n (%)			
Red	848 (84)	1170 (85)	2018 (85)
Orange	3459 (70)	4491 (71)	7950 (70)
Yellow	13,835 (65)	14,771 (63)	28,606 (64)
Green	5304 (59)	4124 (56)	9428 (58)
Time to see physician in minutes, median (IQR)	50 (71)^b^	55 (73)^c^	53 (72)^d^
Length of stay (LOS) in minutes, median (IQR)	153 (119)	180 (140)	166 (131)
Discharged n (%)			
Home	19,729 (54)	22,928 (60)	42,657 (57)
General ward	16,202 (45)	15,165 (39)	31,367 (42)
Intensive care unit	326 (0.9)	303 (0.8)	629 (0.8)
ED mortality	26 (0.1)	23 (0.1)	49 (0.1)
LWBS^e^	40 (0.1)	103 (0.3)	143 (0.2)

^a^With and without referral

^b^Missing 401

^c^Missing 1178

^d^Missing 1579

^e^LWBS, left without being seen by a physician

### The ability of RETTS© to identify patients at risk for ten-day mortality or ICU admission over time

**Fig. 3:**
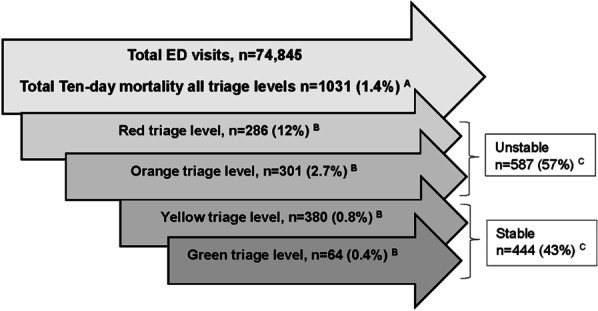
Ten-day mortality in total, crude data ^A^Percentage from the total of ED visits. ^B^Percentage per triage level. ^C^Percentage of ten-day mortality

In total, 1031 (1.4%) of all patients died within ten days after the ED visit (Fig. 3). The majority of these patients were assessed as unstable upon arrival to the ED, n = 587 (57%). The remaining patients (n = 444; 43%) were assessed as stable, i.e. allocated a yellow or green triage level. The risk for ten-day mortality decreased from 12% in the red triage level, to less than 1% in the yellow and green triage levels.

As depicted in Table 2, ten-day mortality and admission to ICU demonstrated statistically significant differences between each triage level compared to the reference in both annual cohorts. Thus, patients allocated red, orange and yellow triage levels had a significantly higher risk of mortality within ten days, or being admitted to an ICU, compared to those patients in the green triage level. The OR declined and CI decreased over time in all triage levels, except for the red triage level considering admission to an ICU. Statistically significant differences were found between the two annual cohorts for ICU admission, but not for ten-day mortality.

**Table 2.**
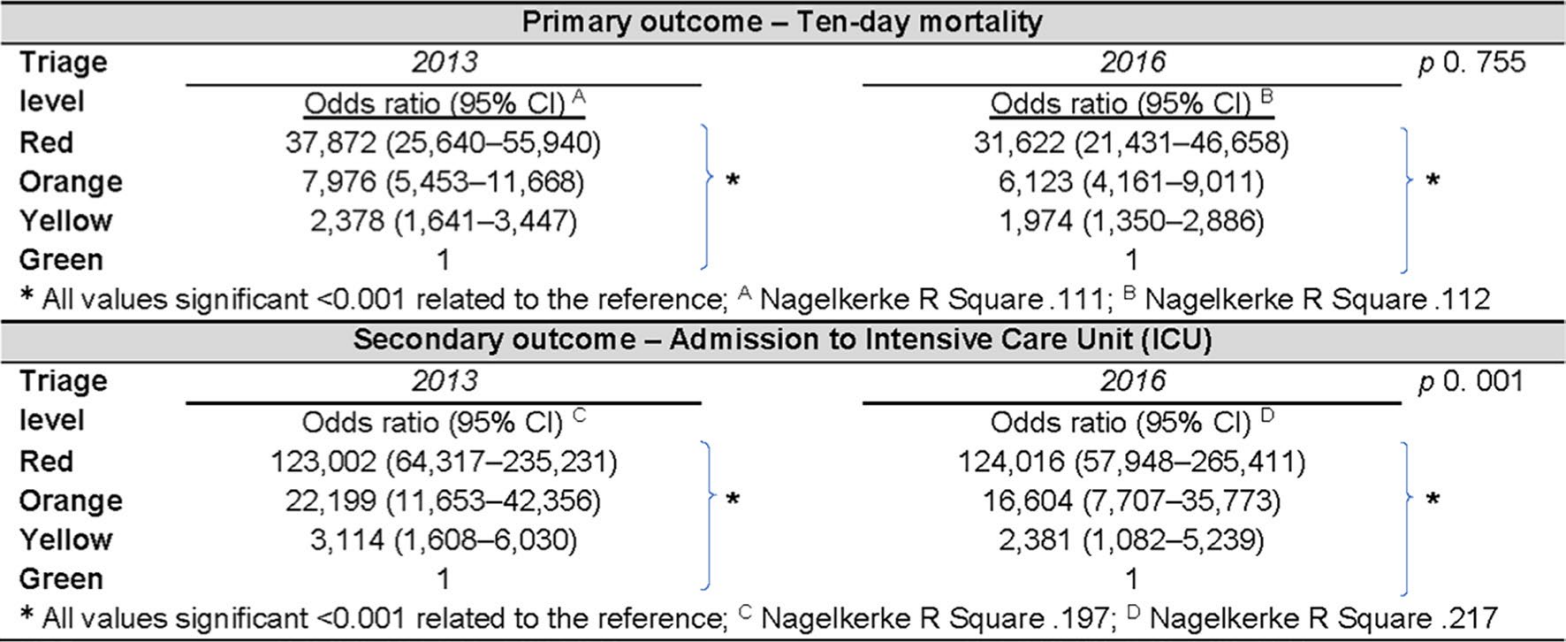
The effect of triage level on primary and secondary outcomes, crude data, multiple logistic regression

### The impact of predictors added to RETTS©

When adjusting for ACCI and gender, a statistically significant difference between the two annual cohorts was seen for ten-day mortality also (Table 3). The OR declined and CI decreased, i.e. the risk of ten-day mortality after the ED visit per triage level was lower in both annual cohorts when adding gender and ACCI, with the largest effect in the 2016 cohort. The opposite was displayed for ICU admission. Adding ACCI and gender yielded an increase in OR and wider CI in both annual cohorts, i.e. the risk of ICU admission increased.

**Table 3.**
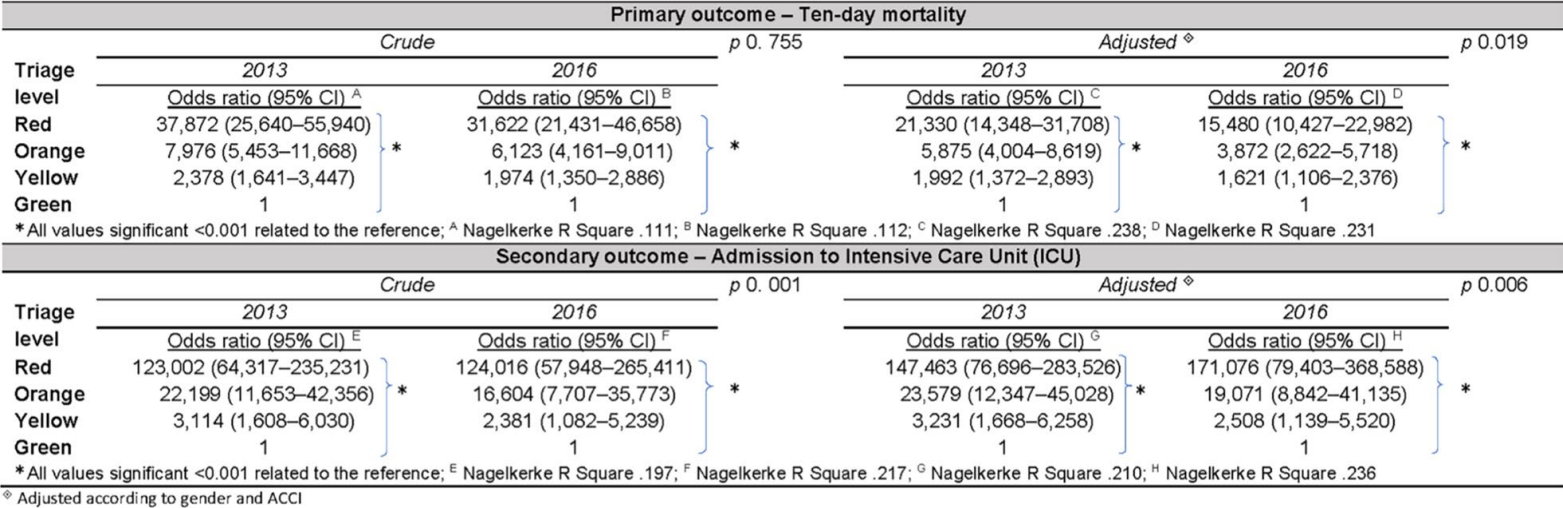
The effect of triage level on primary and secondary outcomes, crude and adjusted per year, multiple logistic regression

**Fig.4:**
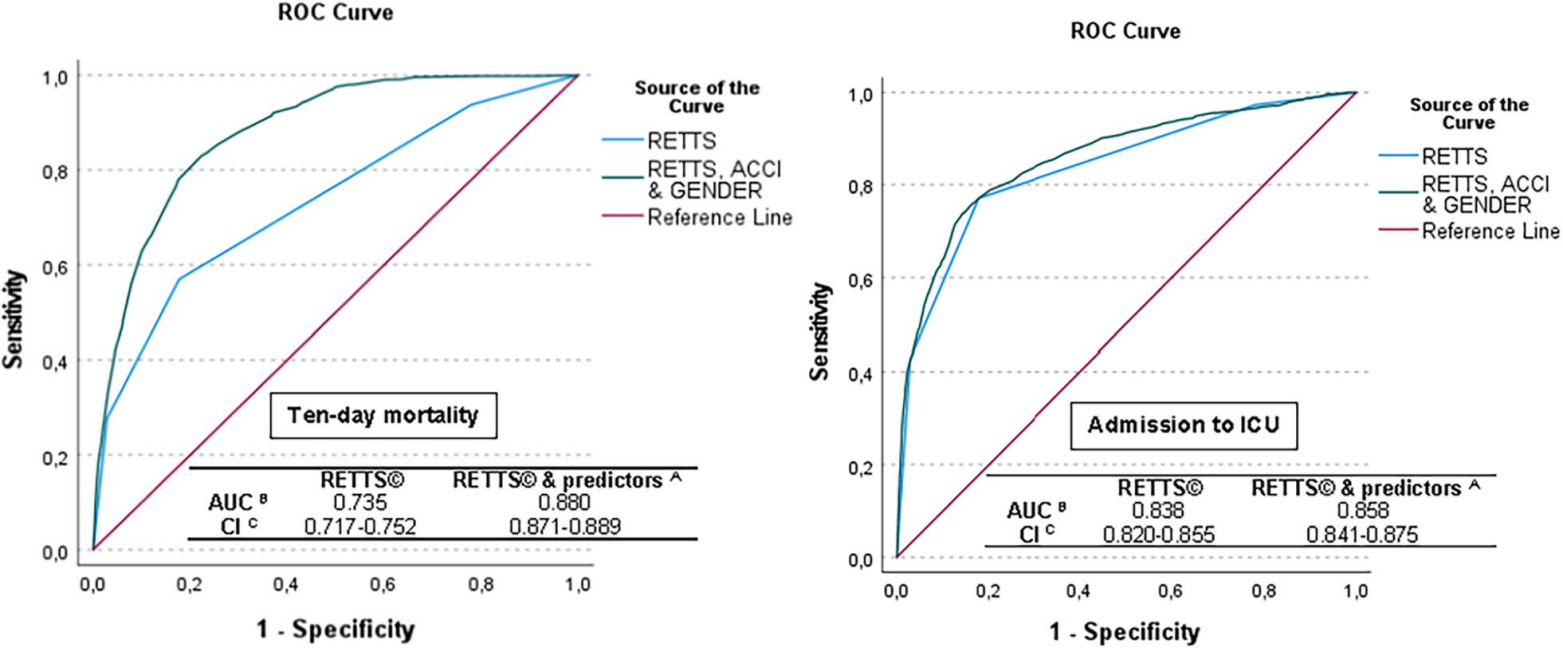
The effect of triage, gender and ACCI on ten-day mortality and ICU admission, 2013 and 2016 together

The impact of RETTS©, gender and ACCI on ten-day mortality and ICU admission is illustrated by ROC curves calculated on all patients together (Fig. 4). The AUC value considering ten-day mortality for the RETTS© triage system alone was 0.735, which indicates an acceptable predictive ability for detecting patients at risk of ten-day mortality. By adding ACCI, AUC increased to 0.878, where age alone generated an AUC value of 0.873. When gender was added, the AUC value increased to 0.880, indicating excellent predictive ability. Regarding ICU admission, the AUC value for RETTS© alone was 0.838, which is excellent. The AUC increased to 0.855 when adding ACCI, where the co-morbidity had a higher impact than age, with an AUC of 0.837. When gender was added to RETTS© and ACCI, the AUC further increased to 0.858.

## Discussion

The main results of this study demonstrate that the annual upgrade of RETTS© had no statistically significant impact on the validity of the triage system, at least for ten-day mortality. However, the results demonstrated the decisive impact of age and co-morbidity, with statistical significance in both outcomes, i.e. both ten-day mortality and admission to an ICU.

In the primary outcome, ten-day mortality, the OR and CI decreased in the adjusted data, meaning morbidity and higher age explain mortality more than RETTS©. However, the opposite difference is displayed in the ICU admission, which showed an increase in OR and CI, particularly on the red triage level. This increase can be interpreted to mean that ICU admission is more a result of the event and/or morbidity, rather than of high age, considering the AUC values. For both annual cohorts, both ten-day mortality and ICU admission, the OR is highest in the red triage level. Regarding the fact that the median age in this study was 61 years, a large number of patients had two ACCI points from the start, without considering any comorbidity. This might explain the effect on the outcomes. Age has been discussed as a predictor to be considered in triage earlier in a study where the discriminative ability of the triage system decreased when the patient’s age increased [28]. Interestingly, in a Swedish study of associations between VS and mortality using RETTS, a side finding was that age was an independent risk factor for mortality [29]. This risk was confirmed by Ruge et al. [30], who identified a strong association with mortality in all triage levels defined by RETTS©, defining older as patients > 60 years. The results of this study confirm that age is a major predictor for outcomes in RETTS© and should be taken into consideration when revising the triage scale again.

Regarding the validity of RETTS© in ED context, the study by Widgren et al. [23] is the most cited. However, the effect of co-morbidity was not investigated and age was reported as descriptive information associated with arrival mode and per triage levels. The study also demonstrated a total mortality rate of 5.2%, which is noticeably higher than the 1.4% in the present study. However, this difference might be associated with different measurements; ten-day mortality versus in-hospital mortality. Nevertheless, Widgren et al. demonstrated statistically significant differences between triage levels and in-hospital mortality [23], which is comparable to the results of the present study. A comparison with Pérez et al. [20] is not completely feasible since they applied outcomes such as admission to a general ward, prolonged in-hospital LOS, and four different mortality rates—none of them ten days. The choice of primary outcome differs with regards to time perspective compared to many other ED triage studies [20, 31]. The reason to use 10-day mortality stems from ED research in which it was found that the majority of adverse events after an ED visit occurs within ten days [32], which a Swedish study confirmed by demonstrating that one third of short time mortality occurred between the eighth and tenth day after the ED visit [33]. However, Pérez et al. did adjust their data, but applied a version of the Charlson index, without age, scoring the patients from zero to 2+ [20]. However, both studies [20, 23] demonstrate a statistical significance between triage levels, which corresponds well with the present study.

In summary, it is difficult to compare validity studies, mainly because there is a lack of consensus about how to define the validity of triage scales and systems, and what outcomes to use [34, 35], as well as the range of study design, study samples and reference standards, which makes comparison even more difficult [36]. One commonly used proxy is over and under triage. Over triage is defined as non-urgent patients who are incorrectly classified as urgent [37], which can result in the inappropriate use of resources, and can potentially have an adverse impact on other patients [38]. Under triage is defined as patients allocated with lower triage levels than their needs require, leading to prolonged waiting time to being assessed by a physician, and therefore increased risk of adverse outcomes [39]. However, there is no consensus about how high degree of under respectively over triage is acceptable. Several studies use the American College of Surgeons Committee on Trauma (ACS COT) as a guideline with figures for acceptable under triage ranging from 1 to 10% and over triage ranging from 25 to 50% [37, 39–41]. The results of this study demonstrate that almost one fifth (n = 13,658) of all patients (n = 74,845) were assessed as unstable, i.e. were assessed as having a life-threatening or potentially life-threatening condition. However, only 587 patients (4.3%) died within ten days, which might be considered as over triage. Furthermore, since there are no guidelines for the ED context about over or under triage, it is difficult to determine. Nevertheless, to some extent, over or under triage is probably needed in order to ensure that no patients will be missed, but has the disadvantage that overuse can reduce compliance with the system [22].

However, according to van Veen and Moll [42], the validity of a triage system is determined by its reliability and ability to predict the true level urgency. Further, its reliability depends on how uniform and complete it is, and how it is understood and applied. Good training and instructions can optimise the use and interpretation of the triage system [42]. Previous studies of RETTS© have demonstrated problems with both education [18] and reliability [43], which may impact the validity of RETTS©.

### Strengths and limitations

The major strength of this study is the adjustment of data according to ACCI in the data analysis around the multiple logistic regression analyses. Another strength is the adjustment of gender. The rather big sample size of 74,845 is another strength. However, the low number of patients who were actually included in the analyses of ten-day mortality and ICU admission may explain the wide CI values, which can be considered as a limitation. The movements of just a few patients between the cohorts can generate a significantly impact on the OR. Hence, the wide CI values warrant caution when interpreting the results. Another limitation is that the study is restricted to two EDs, which might limit the generalisability of the study.

A selection bias is inevitable when patients ≤ 18 years are excluded, but also when considering the fact that elderly patients are rarely admitted to an ICU. The lack of consensus about how to validate a triage scale can be considered as a limitation that affects all validity studies [34]. The use of different references [35] and different ED contexts [44] as well as mortality rates [20, 31, 32], make comparisons with other studies difficult.

## Conclusions

The results of this study demonstrate that the annual upgrade of RETTS© had no statistically significant impact on the validity of the triage system, at least with regards to the risk of dying within 10 days of an ED visit. However, the inclusion of ACCI, or at least age, can improve the validity of RETTS©. Furthermore, the results show a statistical significance between triage levels, i.e. that there is a statistically higher risk of both ten-day mortality and ICU admission for all triage levels compared to the green triage level, whether or not adjusted for gender and ACCI. Finally, the results demonstrate a suspected over triage. However, it is difficult to evaluate as there is no consensus on over or under triage in an ED context.

## Data Availability

The dataset generated during and/or analysed during the current study are not publicly available due to the ethical approval but are available from the corresponding author on reasonable request.

## Appendix

A longitudinal, retrospective registry-based validation study of RETTS©, the Swedish adult ED context version.

**Table:**

Age	Points
Age-combined co-morbidity index, according to Charlson et al. [25]	
41–49 years	0 points
50–59 years	1point
60–69 years	2 points
70–79 years	3 points
80–89 years	4 points
≥ 90 (5p)	5 points

Disease	ICD-10 code
Charlson co-morbidity Index ICD 10 version according to Sundararajan et al. [26]	
Acute myocardial infarction (1p)	I21, I22, I252
Congestive heart failure (1p)	I50
Peripheral vascular disease (1p)	I71, I790, I739, R02, Z958, Z959
Cerebrovascular insult (1p)	I60, I61, I62, I63, I65, I66, G450, G451, G452, G458, G459, G46, I64, G454, I670, I671, I672, I674, I675, I676, I677 I678, I679, I681, I682, I688, I69
Dementia (1p)	F00, F01, F02, F051
Pulmonary disease (1p)	J40, J41, J42, J44, J43, J45, J46, J47, J67, J60, J61, J62, J63, J66, J64, J65
Connective tissue disease (1p)	M32, M34, M332, M053, M058, M059, M060, M063, M069, M050, M052, M051, M353
Gastric ulcer (1p)	K25, K26, K27, K28
Hepatic disease (1p)	K702, K703, K73, K717, K740, K742, K746, K743, K744, K745
Diabetes mellitus (1p)	E109, E119, E139, E149, E101, E111, E131, E141, E105, E115, E135, E145
Complications of diabetes mellitus (2p)	E102, E112, E132, E142 E103, E113, E133, E143 E104, E114, E134, E144
Paraplegia (2p)	G81 G041, G820, G821, G822
Renal disease (2p)	N03, N052, N053, N054, N055, N056, N072, N073, N074, N01, N18, N19, N25
Cancer (2p)	C0, C1, C2, C3, C40, C41, C43, C45, C46, C47, C48, C49, C5, C6, C70, C71, C72, C73, C74, C75, C76, C80, C81, C82, C83, C84, C85, C883, C887, C889, C900, C901, C91, C92, C93, C940, C941, C942, C943, C9451, C947, C95, C96
Metastatic cancer (3p)	C77, C78, C79, C80
Advanced hepatic disease (3p)	K729, K766, K767, K721
HIV (6p)	B20, B21, B22, B23, B24

## Abbreviations

ACCI: Age Combined Comorbidity Index
ADAPT: Adaptive process triage
ATS: Australasian Triage Scale
AUC: Area under curve
CI: Confidence Interval
CTAS: Canadian Triage and Acuity Scale
DEPT: Danish emergency process triage
ED: Emergency Department
ESI: Emergency Severity Index
ESS: Emergency symptoms and signs
ICD: International Statistical Classification of Diseases and Related Health Problems
ICU: Intensive Care Unit
LOS: Length of stay
LWBS: Left without being seen
METTS: Medical emergency triage and treatment system
MTS: Manchester triage system
OR: Odds ratio
RETTS©: Rapid emergency triage and treatment system
RETTS-HEV: Rapid Emergency Triage and Treatment System—Hospital Unit West
RN: Registered nurse
ROC: Receiver operating characteristics
SBU: The Swedish Council of Health Technology Assessment
VS: Vital signs
